# The CB2 Agonist β-Caryophyllene in Male and Female Rats Exposed to a Model of Persistent Inflammatory Pain

**DOI:** 10.3389/fnins.2020.00850

**Published:** 2020-08-18

**Authors:** Ilaria Ceccarelli, Paolo Fiorenzani, Federica Pessina, Jessica Pinassi, Margherita Aglianò, Vincenzo Miragliotta, Anna Maria Aloisi

**Affiliations:** ^1^Department of Medicine, Surgery and Neuroscience, University of Siena, Siena, Italy; ^2^Department of Molecular and Developmental Medicine, University of Siena, Siena, Italy; ^3^Department of Veterinary Sciences, University of Pisa, Pisa, Italy

**Keywords:** cannabinoids, sex differences, formalin test, rats, persistent pain

## Abstract

Cannabinoids help in pain treatment through their action on CB1 and CB2 receptors. β-caryophyllene (BCP), an ancient remedy to treat pain, is a sesquiterpene found in large amounts in the essential oils of various spice and food plants such as oregano, cinnamon, and black pepper. It binds to the CB2 receptor, acting as a full agonist. Sex differences in the BCP-induced analgesic effect were studied by exposing male and female rats to a persistent/repeated painful stimulation. To simulate treatment of a repeated inflammatory condition, after the first formalin injection (FT1; 50 μl, 2.5%), rats received BCP *per os* for 7 days at two dosages: 5 and 10 mg/kg dissolved in olive oil (OIL). The control group was treated with OIL for 7 days. On day 8, the formalin test was repeated (FT2) with a lower formalin concentration (50 μl, 1%). During the first and second formalin tests, pain-induced responses (licking, flexing, and paw jerk) and spontaneous behaviors were recorded and analyzed. In the FT1 (before the beginning of treatment with BCP), females displayed higher pain responses than did males in terms of flexing duration during the first part of the test (I phase and interphase), while during the second part (II phase early and late) males showed higher levels than did females in licking duration. In the FT2, the pain responses generally decreased in the BCP groups in a dose-dependent manner (i.e., greater effect of BCP10), with a more pronounced reduction in males than in females; moreover, the pain responses remained high in the OIL groups and in the female BCP5 group. In conclusion, long-term intake of BCP appears to be able to decrease pain behaviors in a model of repeated inflammatory pain in both sexes, but to a greater degree in males.

## Introduction

In Europe, the number of chronic pain patients is approaching 50% of the population, most of them suffering pain daily and at high intensity. Chronic pain is often difficult to treat and can be very disabling (Gatchel et al., 2014). Pain therapists and patients have accepted the inclusion of analgesic substances known to have serious side effects, i.e., opioids, in the list of “easily-prescribable” drugs. We have repeatedly shown that opioids, and analgesics in general, can induce hypogonadism, a persistent and serious side effect (Ceccarelli et al., 2006; Aloisi et al., 2009; Pergolizzi et al., 2010). This condition impairs the nervous system (depression) as well as muscle tone (asthenia and fatigue), leading to further chronic pain (De Maddalena et al., 2012). Therefore, increased attention has been given to other plant-based compounds with less severe consequences for the patient’s body.

An important example is cannabis. The presence in the body of a specific cannabinoid system with receptors (CB1 and CB2) and ligands (anandamide), often related to pain pathways and pain modulatory structures, prompted the use of preparations with these active ingredients. Nevertheless, the role of the cannabis plant and its components (cannabinoids) as adjuvant analgesics in the treatment of chronic pain has been the subject of long-standing controversy (Starowicz and Finn, 2017; Woodhams et al., 2017).

The analgesic effect of cannabis (the exogenous ligand) in preclinical and clinical studies at both central and peripheral levels is well known, and it is commonly used in chronic pain treatment, although the results are not always constant (Ashton and Milligan, 2008; Hosking and Zajicek, 2008; Prospéro-García et al., 2020).

The cannabinoid receptor CB1 is known to be distributed mainly in the central nervous system (CNS) and is considered responsible for the psychotropic effect (Witkin et al., 2005). The CB2 receptor is also expressed in the CNS and in immune cells, but its stimulation does not induce psychotropic effects (Galiègue et al., 1995; Galve-Roperh et al., 2006; Ofek et al., 2006; Zhang et al., 2007). Plant extracts can include substances able to interact with these receptors. β-caryophyllene (BCP, an FDA-approved food additive), present in many plants such as oregano, cinnamon, and cloves, is a selective ligand for CB2 and acts as a full agonist (Zheng et al., 1992; Mockute et al., 2001; Jayaprakasha et al., 2003; Gertsch et al., 2008; Brizzi and Pessina, 2018). Its ability to decrease pain was shown in *male* rats and mice with single or repeated administration (Fiorenzani et al., 2014; Klauke et al., 2014; Aly et al., 2019).

Males and females differ in many aspects of pain, from molecular to behavioral levels. In humans, many chronic pain syndromes are more common in females, while others are more common in males (Pieretti et al., 2016); genes, hormones, and epigenetics have been invoked to explain these differences (Aloisi and Bonifazi, 2006; Sorge and Totsch, 2017). In experimental models, sex differences change (higher or lower in one sex) depending on methodological factors, such as the kind of painful stimulation (mechanical vs. electric) or the intensity of the chemical stimulation (formalin concentration 10 vs. 1%) (Aloisi et al., 1995; Craft et al., 2004).

The cannabinoid system has been studied in both sexes in humans and experimental animals, with the results showing sex differences depending on the dose used and the parameter taken into consideration (Craft and Leitl, 2008; Diaz et al., 2009; Craft et al., 2013).

The aim of the present experiment was to evaluate the analgesic effect of β-caryophyllene (CB2 agonist) in male and female rats with a model of repeated formalin injection. The formalin test was carried out twice with 1 week in between, allowing the determination of the long-lasting effects of the first painful stimulus and the possible modulatory effect of a CB2 agonist.

## Materials and Methods

The study was carried out on male and female Wistar rats (Harlan-Nossan, Milan, Italy), weighing 225–250 and 200–225 g, respectively, at their arrival. The animals were housed two per cage in plastic-bottomed cages with sawdust bedding; they were separated by a transparent Plexiglas wall with holes to allow social interaction and to avoid physical contact during the testing period. Cages were kept at room temperature of 21 ± 1°C, relative humidity of 60 ± 10%, and on a 12/12 h light/dark cycle (lights off at 7 a.m.). They received food and water *ad libitum*.

All experimental tests were carried out during the active period of the rodents between 09:30 and 12:30 a.m., in a dedicated room, under red light and white noise. Attention was paid to the regulations for handling laboratory animals of the European Communities Council Directive (86/609/EEC) and the ethical guidelines for the investigation of experimental pain in conscious animals issued by the *ad hoc* Committee of the International Association for the Study of Pain (Zimmermann, 1983). This study received the approval of the Local Ethics Committee (Aut Min 65/2011B). Particular efforts were made to minimize animal suffering and to reduce the number of animals used.

### Experimental Schedule

**FIGURE 1:**
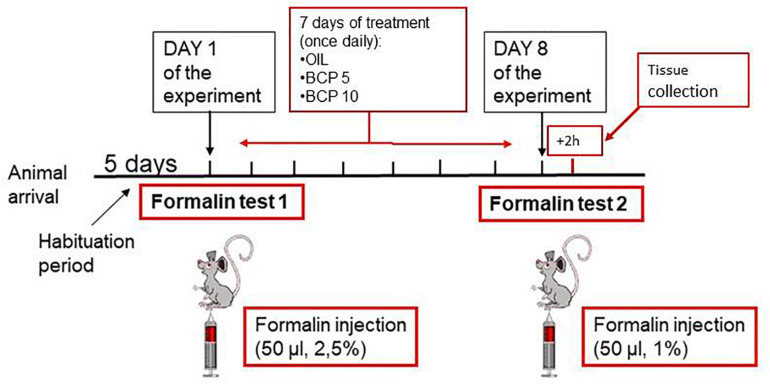
Schematic representation of the experimental design: on the day of the experiment, the two animals belonging to a cage were subjected to the first formalin test (FT1) together in two separate open fields. The formalin concentration was 2.5%. At the end of FT1, the animals were reintroduced into their home cage and randomly assigned to treatment groups. Vehicle or substance [olive oil (OIL), β-caryophyllene (BCP) 5 mg/kg, or BCP 10 mg/kg] was given by oral gavage once a day (at 4 p.m.) from days 1 to 7. On day 8, the animals underwent the second formalin test (FT2) at a lower formalin concentration (1%). Two hours after FT2, the animals were deeply anesthetized and intracardially perfused to collect tissues for histological examinations.

As schematically reported in Figure 1, all animals underwent two formalin tests (FT1 on day 1 and FT2 on day 8) separated by a 1-week period in which each animal received the tested compounds daily (days 1–7).

### Experimental Procedure

On the day of the experiment, the two animals belonging to a cage were transported to the experimental room to be subjected together to the formalin test in two separate open fields. At the end of FT1, the animals were reintroduced into their home cage and randomly assigned to treatment groups. Vehicle (olive oil, OIL) or β-caryophyllene (BCP in OIL) was given by oral gavage once a day (at 4 p.m.) from days 1 to 7; the volume of administration was based on the body weight (1 ml/kg), recorded daily. The formalin concentration was different between the first and second tests: FT1, 2.5%; FT2, 1%.

Animals were allocated to the following experimental groups:

• Olive oil used as vehicle (OIL, nine males, nine females);
• BCP 5.0 mg kg^–1^ day^–1^ (BCP5, nine males and nine females);
• BCP 10.0 mg kg^–1^ day^–1^ (BCP10, nine males and eight females).

#### Formalin Test (FT1 and FT2)

The formalin test allows measuring pain intensity in freely moving animals after receiving a painful stimulation (subcutaneous injection in the dorsal right hind paw of 50 μl of a dilute solution of freshly prepared formalin). Immediately after the injection, the rat was placed in an open-field apparatus where spontaneous and pain-evoked behavioral responses were recorded for 60 min by a video camera and analyzed in 12 periods of 5 min. The responses were divided into four phases to better underline the time course of the events, as follows:

I phase: 0–10 min (periods 1 and 2);
Interphase: 10–20 min (periods 3 and 4);
II phase early: 20–40 min (periods 5, 6, 7, and 8);
II phase late: 40–60 min (periods 9, 10, 11, and 12).

The following behavioral responses were considered:

• Formalin-induced responses: licking duration (time spent licking the injected foot); flexing duration (time spent with the leg held off the floor, flexed close to the body); and paw jerk frequency (number of phasic flexions of the leg). These behavioral responses are indicative of different neural circuits, from the most spinal-mediated (paw jerking) to the more supraspinal-mediated (licking).
• Spontaneous behavior: activity duration (time spent sniffing and looking around the environment); rearing frequency (number of times the animal stood on its fore limbs); grooming duration (time spent licking and scratching the body); sit alert duration (time spent motionless but in an alert posture); and crouch duration (time spent motionless in a sleep-like position).

#### Tissue Collection

Two hours after FT2, the animals were deeply anesthetized (sodium pentobarbital > 70 mg/kg body weight) and intracardially perfused with phosphate-buffered saline (PBS, about 300 ml) for exsanguination of the tissues. Then, the gastrointestinal tract and the skin of the injected paw were collected and stored in 4% paraformaldehyde for histological examination to evaluate the formalin injection site lesion and potential toxic gastrointestinal effects due to oral administration of the test compound. After formalin fixation, the samples were paraffin embedded and the sections were hematoxylin and eosin stained. Skin lesions were graded as minimal, mild, moderate, or severe for edema and inflammation. Inflammatory infiltrate was defined as neutrophilic, lymphoplasmacellular, or mixed.

### Statistical Analysis

Data are reported as the mean and SEM in the tables and figures. ANOVA was carried out to determine the following:

Step 1: Sex differences in spontaneous and formalin-induced behaviors during FT1,
Step 2: Sex differences in spontaneous and formalin-induced behaviors during FT2,
Step 3a: Sex differences in the pain responses between FT1 and FT2, repeated,
Step 3b: Sex differences in the percentage of changes in pain responses between FT1 and FT2, repeated.

Depending on the step, comparisons were carried out with the factors sex (two levels: male and female); phase (four levels: I phase, interphase, II phase early, and II phase late, singly or repeated); treatment (three levels: OIL, BCP5, and BCP10); and test (two levels: FT1 and FT2). Fisher’s least square deviation (LSD) test was used as *post hoc* analysis when necessary. *P* < 0.05 was considered significant.

## Results

No signs of toxicity or discomfort were observed in male and female rats throughout the experiment. Out of the 27 males and 26 females, behavioral data from three males and one female were lost during FT2.

### Body Weight

Two way ANOVA applied to body weight values (recorded daily in all animals) with the factors sex (two levels: males and females) and days (eight levels: days 1–8, repeated) revealed an effect of sex [*F*(1, 43) = 90.50, *p* = 0.001] and a significant interaction of sex × days [*F*(7, 301) = 6.55, *p* = 0.001] due to the heavier weight of males than females (285 ± 15 g vs. 248 ± 18 g) and the progressive increase from days 1 to 8 in males, but not in females.

### Step 1: Formalin Test (FT1) in Male and Female Rats

**FIGURE 2:**
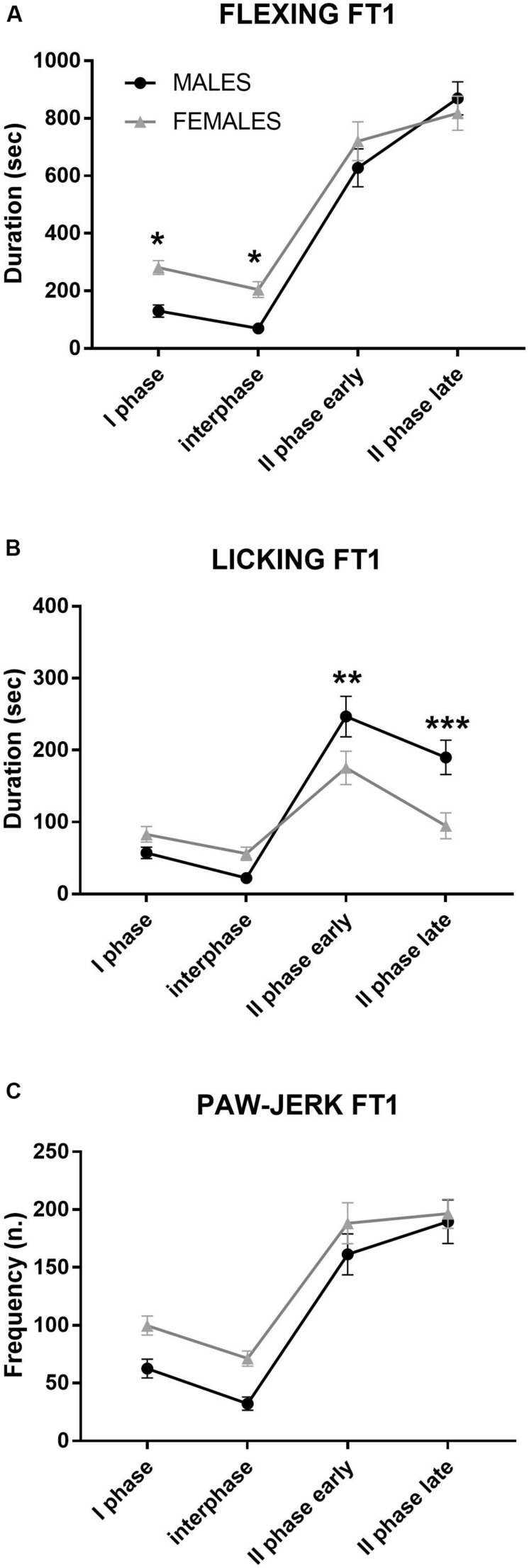
Formalin test 1 (FT1): differences between males and females in formalin-induced flexing **(A)**, licking **(B)**, and paw jerk **(C)** during the 60 min of the test. For clarity, the time course was subdivided into four phases: I phase, 0–10 min; interphase, 10–20 min; II phase early, 20–40 min; and II phase late, 40–60 min. *p < 0.05, **p < 0.01, and ***p < 0.001 vs. other sex, same phase. Values are the mean ± SEM.

As reported in Table 1 for spontaneous behaviors and Figure 2 and Table 2 for formalin-induced behavioral responses, data from male and female rats recorded during the FT1 were analyzed by two-way ANOVA with the factors sex (two levels: male and female) and phase (four levels: I phase, interphase, II phase early, and II phase late, repeated). Since at this point the animals were not yet divided into treatment groups, all males (*n* = 27) were compared with all females (*n* = 26). Formalin injection (50 μl, 2.5%) induced pain behaviors in all animals. As shown in Table 1, locomotion duration and rearing frequency (measures of activity) were significantly higher in males than in females independently of the phases. Crouch duration, a measure of immobility, was higher in females II phase (early and late).

**TABLE 1:** Spontaneous behaviors recorded during the first (FT1) and the second (FT2) formalin test means ± SEM.

	**Formalin test 1**					**Formalin test 2**			
	**I phase**	**Interphase**	**II phase early**	**II phase late**		**I phase**	**Interphase**	**II phase early**	**II phase late**
**Locomotion (s) FT1: sex: *F*(1, 51) = 3.9, *p* = 0.05 males > females**									
Male (*N* = 27)	354.3 ± 22.4	230.4 ± 24.4	252.7 ± 44.1	174.6 ± 44.5	OIL (*N* = 9)	291.7 ± 31.3	143.4 ± 31.0	234.4 ± 50.9	252.1 ± 54.7
					BCP5 (*N* = 8)	397.1 ± 40.3	210.0 ± 50.1	186.4 ± 76.2	224.1 ± 98.4
					BCP10 (*N* = 7)	395.1 ± 36.8	210.6 ± 66.3	336.6 ± 81.7	142.7 ± 67.9
Female (*N* = 26)	304.7 ± 18.1	186.8 ± 22.1	174.8 ± 23.0	99.6 ± 29.6	OIL (*N* = 9)	335.6 ± 29.2	173.0 ± 44.7	161.6 ± 36.1	142.7 ± 30.9
					BCP5 (*N* = 8)	338.9 ± 32.6	181.4 ± 48.3	113.6 ± 34.2	200.3 ± 62.4
					BCP10 (*N* = 8)	238.1 ± 39.1	170.1 ± 39.8	167.8 ± 84.4	139.8 ± 69.4
**Sit alert (s)**									
Male (*N* = 27)	90.5 ± 11.9	116.6 ± 15.5	99.1 ± 12.9	65.1 ± 11.0	OIL (*N* = 9)	117.9 ± 24.2	113.3 ± 28.8	144.3 ± 51.1	184.8 ± 56.0
					BCP5 (*N* = 8)	85.4 ± 18.1	117.9 ± 31.4	105.3 ± 32.7	138.0 ± 49.6
					BCP10 (*N* = 7)	149.1 ± 43.8	247.6 ± 80.4	332.1 ± 67.6	304.0 ± 132.1
Female (*N* = 26)	82.2 ± 11.9	101.6 ± 18.2	72.0 ± 15.0	54.5 ± 14.8	OIL (*N* = 9)	118.8 ± 30.3	121.7 ± 16.6	196.3 ± 29.4	162.7 ± 29.1
					BCP5 (*N* = 8)	127.1 ± 28.5	211.9 ± 50.6	169.0 ± 59.4	159.5 ± 42.5
					BCP10 (*N* = 8)	157.5 ± 29.2	185.4 ± 40.8	202.8 ± 65.3	206.4 ± 36.4
**Grooming (s)**									
Male (*N* = 27)	37.6 ± 5.8	43.9 ± 7.4	104.6 ± 10.5	67.8 ± 13.1	OIL (*N* = 9)	44.1 ± 10.5	61.2 ± 13.4	164.7 ± 28.6	110.6 ± 21.6
					BCP5 (*N* = 8)	45.6 ± 7.7	113.9 ± 29.2	112.6 ± 21.2	80.3 ± 28.3
					BCP10 (*N* = 7)	15.0 ± 6.2	40.1 ± 17.8	100.7 ± 33.2	128.9 ± 51.7
Female (*N* = 26)	52.7 ± 10.1	62.4 ± 8.3	99.3 ± 11.3	85.3 ± 17.0	OIL (*N* = 9)	38.7 ± 12.4	77.6 ± 17.2	117.0 ± 21.9	55.4 ± 23.8
					BCP5 (*N* = 8)	64.5 ± 26.4	61.9 ± 15.7	129.3 ± 22.2	75.3 ± 25.5
					BCP10 (*N* = 8)	59.8 ± 11.1	81.9 ± 14.2	142.4 ± 16.2	111.0 ± 21.7
Crouch (s)FT1: sex × phase: *F*(3, 153) = 4.1, *p* = 0.007; **p < 0.01 vs. other sex, same phase									
Male (*N* = 27)	48.1 ± 13.7	172.9 ± 25.9	490.3 ± 43.4	696.3 ± 48.2	OIL (*N* = 9)	80.7 ± 27.0	223.3 ± 47.0	456.8 ± 93.3	571.1 ± 64.4
					BCP5 (*N* = 8)	28.8 ± 20.5	149.6 ± 62.4	720.6 ± 114.8	730.4 ± 154.4
					BCP10 (*N* = 7)	13.4 ± 7.5	94.9 ± 54.1	390.9 ± 106.4	591.1 ± 132.9
Female (*N* = 26)	70.1 ± 15.6	189.2 ± 25.6	678.3 ± 32.5**	865.6 ± 59.1**	OIL (*N* = 9)	14.0 ± 11.8	130.3 ± 47.8	421.3 ± 63.9	663.3 ± 71.9
					BCP5 (*N* = 8)	16.1 ± 11.8	110.1 ± 38.6	526.8 ± 103.1	700.5 ± 75.8
					BCP10 (*N* = 8)	91.3 ± 43.6	144.3 ± 56.8	548.0 ± 113.0	698.3 ± 113.8
**Rearing (*n*)FT1: sex: *F*(1, 50) = 7.79, *p* = 0.007 males > females**									
Male (*N* = 27)	34.7 ± 3.3	15.1 ± 2.6	14.0 ± 3.7	6.5 ± 2.0	OIL (*N* = 9)	20.7 ± 2.6	6.4 ± 2.1	14.1 ± 4.2	11.7 ± 5.0
					BCP5 (*N* = 8)	32.6 ± 5.4	14.5 ± 4.9	10.1 ± 4.3	20.3 ± 9.6
					BCP10 (*N* = 7)	33.1 ± 7.1	11.6 ± 4.2	13.1 ± 5.0	8.6 ± 4.3
Female (*N* = 26)	22.6 ± 2.1	9.6 ± 1.7	5.0 ± 2.1	2.8 ± 0.9	OIL (*N* = 9)	29.7 ± 4.4	18.2 ± 5.9	11.2 ± 2.9	14.6 ± 4.0
					BCP5 (*N* = 8)	31.9 ± 4.8	12.6 ± 4.3	6.0 ± 1.7	12.6 ± 4.8
					BCP10 (*N* = 8)	25.8 ± 6.2	18.4 ± 7.8	16.1 ± 11.4	12.1 ± 7.2

**Two-way ANOVA was carried out during FT1 with factors sex and phase and during FT2 with factors sex, treatment, and test for each phase. The results are included in the table.**

**TABLE 2:** Results of the two-way ANOVA applied to the formalin-induced responses recorded during formalin test 1 (FT1) in male and female rats.

	**Sex*F*(1, 51) =**	**Sex × phase*F*(3, 153) =**	***Post hoc***
Flexing	n.s.	2.75, *p* = 0.04	Females > males I, phase Females > males, interphase
Licking	243.8, *p* = 0.0001	7.76, *p* = 0.001	Males > females, II phase early Males > females, II phase late
Paw jerk	4.52, *p* = 0.03	n.s.	Females > males, independent of phases

**Results of the factor phase were not included. See also Figure 2 for details. n.s., not significant.**

As shown in Figure 2, flexing duration was higher in females than in males during the first half of the test (I phase, interphase), while males showed a longer licking duration during the II phase (early and late).

### Step 2: Formalin Test (FT2) in Male and Female Rats

**FIGURE 3:**
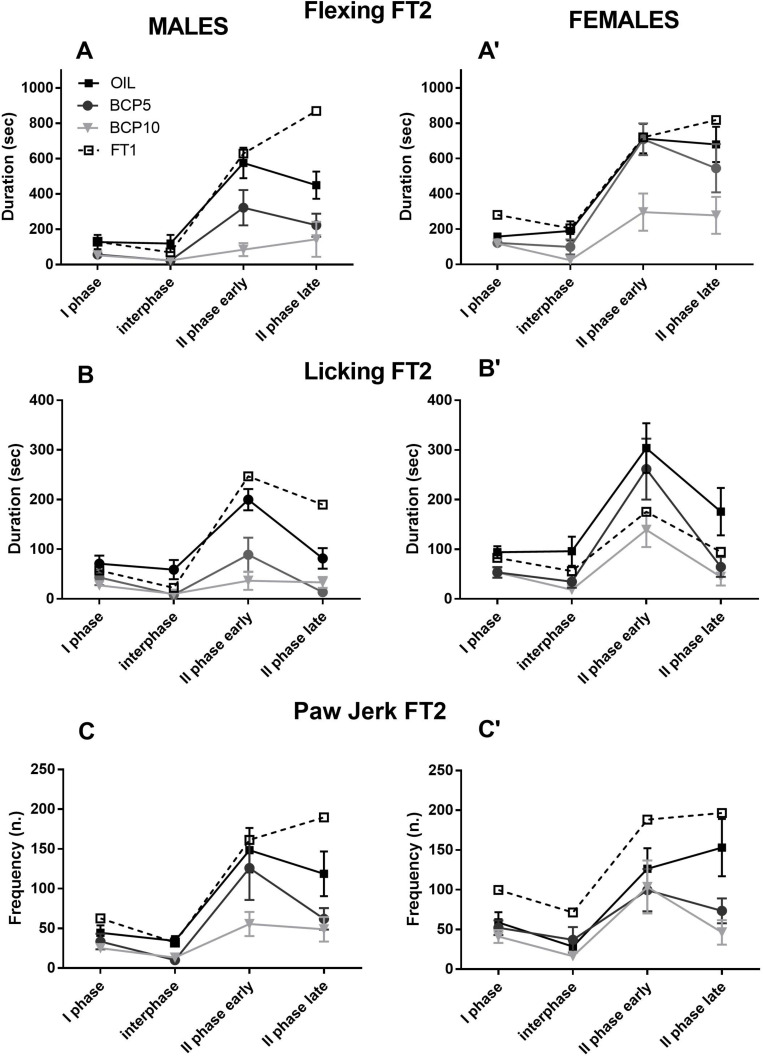
Formalin test 2 (FT2) in olive oil (OIL), β-caryophyllene 5 mg/kg (BCP5), and BCP 10 mg/kg (BCP10) male (*left*) and female (*right*) groups (*dashed line*: FT1 values for comparisons). **(A,A′)** Flexing duration was higher in females than in males during the I and II phases (both early and late: *p* < 0.007, *p* < 0.001, and *p* < 0.007, respectively). BCP5 decreased the flexing duration only in the I phase and interphase (*p* < 0.02 and *p* < 0.01, respectively), whereas BCP10 reduced it in all four phases (*p* < 0.02 for the I phase and *p* < 0.001 for the other phases). **(B,B′)** The licking duration was higher in females than in males only in the II phase early and late (*p* < 0.001 and *p* < 0.01, respectively). BCP5 decreased the licking duration in all phases, except the II phase early (*p* < 0.01, *p* < 0.001, and *p* < 0.001, respectively), whereas BCP10 reduced the licking duration in all four phases (*p* < 0.003 for the I phase and *p* < 0.001 for the other phases). **(C,C′)** The paw jerk frequency was higher in males than in females in the I phase (*p* > 0.05) and lower in BCP5 and BCP10 than in OIL in the II phase late (*p* < 0.001 for both). Values are the mean ± SEM.

As reported in Table 1 for spontaneous behaviors and Figure 3 and Table 3 for formalin-induced behaviors, the data recorded in male and female rats during the FT2 were analyzed by two-way ANOVA with the factors sex (two levels: males and females) and treatment (three levels: OIL, BCP5, and BCP10) for each phase (I phase, interphase, II phase early, and II phase late) in order to better describe the differences in the time course of all behaviors among the three groups for each phase.

**TABLE 3:** Results of the two-way ANOVA applied to the formalin-induced responses recorded during formalin test 2 (FT2).

	**I phase**	**Interphase**	**II phase early**	**II phase late**
Flexing	Sex: *F*(1, 43) = 7.98, *p* = 0.007 Treatment: *F*(2, 43) = 3.92, *p* = 0.02 Females > males OIL > BCP5, BCP10	Sex: n.s. Treatment: *F*(2, 43) = 6.58, *p* = 0.003 OIL > BCP5, BCP10	Sex: *F*(1, 43) = 11.45, *p* = 0.001 Treatment: *F*(2, 42) = 13.61, *p* = 0.001 Females > males OIL, BCP5 > BCP10	Sex: *F*(1, 43) = 7.92, *p* = 0.007 Treatment: *F*(2, 43) = 6.35, *p* = 0.003 Females > males OIL, BCP5 > BCP10
Licking	Sex: n.s. Treatment: *F*(2, 43) = 6.0, *p* = 0.01 OIL > BCP5, BCP10	Sex: n.s. Treatment: *F*(2, 43) = 8.57, *p* = 0.001 OIL > BCP5, BCP10	Sex: *F*(1, 43) = 5.89, *p* = 0.01 Treatment: *F*(2, 43) = 8.26, *p* = 0.001 Females > males OIL, BCP5 > BCP10	Sex: *F*(1, 43) = 5.89, *p* = 5.89 Treatment: *F*(2, 43) = 8.12, *p* = 0.001 Females > males OIL > BCP5, BCP10
Paw jerk	Sex: *F*(1, 43) = 4.26, *p* = 0.04 Treatment: n.s. Males > females	Sex: n.s. Treatment: n.s.	Sex: n.s. Treatment: n.s.	Sex: n.s. Treatment: *F*(2, 43) = 7.8, *p* = 0.001 OIL > BCP5, BCP10

**Results are given per phase. See also Figure 3 for more details. n.s., not significant*.*

As illustrated in Figure 3 and summarized in Table 3, the flexing and licking durations showed differences among groups in all phases due to the BCP groups having lower values than did OIL in both sexes (BCP5 in the I phase and interphase and BCP10 in all phases). Moreover, sex was significant in the I and II phases due to the higher levels in females than in males independently of treatment. Paw jerk frequency was the least affected response; it showed sex differences only in the I phase, with higher levels in males, and a BCP-related decrease in the II phase late.

### Step 3a: Sex Differences in the Pain Responses Between FT1 and FT2

To test the long-term effect of FT1 and the effect of FT2 (after 1 week of OIL or BCP treatment) on the pain responses, three-way ANOVA was carried out with the factors sex (two levels: males and females); treatment (three levels: OIL, BCP5, and BCP10); and test (two levels: FT1 and FT2, repeated) for each phase (I phase, interphase, II phase early, and II phase late). As reported in Figure 3 and Table 4, flexing was decreased from FT1 to FT2 due, in particular, to BCP10 treatment. Licking was higher in females than in males and during both II phases was decreased by treatment, particularly in males. Paw jerk was also decreased by treatment particularly during the second phase in males.

**TABLE 4:** Results of the two-way ANOVA applied to the formalin-induced responses recorded during formalin test 1 (FT1) and formalin test 2 (FT2) with the factors sex, treatment, and test.

	**Formalin test 1 vs. formalin test 2**			
	**I phase**	**Interphase**	**II phase early**	**II phase late**
Flexing	Sex: *F*(1, 42) = 26.0, *p* = 0.01 Treatment: n.s. Test: *F*(1, 42) = 23.57, *p* = 0.001 Sex × test: *F*(1, 42) = 6.73, *p* = 0.01 Females > males in FT1 FT1 > FT2	Sex: *F*(1, 42) = 14.7, *p* = 0.001 Treatment: n.s. Test: *F*(1, 42) = 8.71, *p* = 0.005 Sex × test: *F*(1, 42) = 5.34, *p* = 0.03 Females > males in FT1 FT1 > FT2	Sex: *F*(1, 42) = 7.27, *p* = 0.01 Treatment: *F*(2, 42) = 8.94, *p* = 0.001 Test: *F*(1, 42) = 16.81, *p* = 0.001 Females > males FT1 > FT2 OIL, BCP5 > BCP10	Sex: *F*(1, 42) = 4.21, *p* = 0.04 Treatment: *F*(2, 42) = 6.25, *p* = 0.004 Test: *F*(1, 42) = 48.49, *p* = 0.001 Females > males FT1 > FT2 OIL, BCP5 > BCP10
Licking	Sex: *F*(1, 42) = 8.5, *p* = 0.005 Treatment: n.s. Test: n.s. Females > males	Sex: *F*(1, 42) = 6.44, *p* = 0.01 Treatment: *F*(2, 42) = 9.5, *p* = 0.001 Test: n.s. Females > males OIL > BCP5, BCP10	Sex: n.s. Treatment: *F*(2, 42) = 7.2, *p* = 0.002 Test: *F*(1, 42) = 4.3, *p* = 0.04 Sex × test: *F*(1, 42) = 11.8, *p* = 0.001 Females < males in FT1 Females > males in FT2 OIL > BCP5, BCP10	Sex: n.s. Treatment: n.s. Test: *F*(1, 42) = 13.8, *p* = 0.001 Treatment × test: *F*(2, 42) = 3.6, *p* = 0.03 Sex × test: *F*(1, 42) = 12.3, *p* = 0.001 Females: FT1 = FT2 Males: FT1 > FT2 OIL: FT1 = FT2 BCP5 and BCP10: FT1 > FT2
Paw jerk	Sex: *F*(1, 42) = 8.91, *p* = 0.004 Treatment: n.s. Test: *F*(1, 42) = 34.39, *p* = 0.001 Females > females FT1 > FT2	Sex: *F*(1, 42) = 12.85, *p* = 0.001 Treatment: n.s. Test: *F*(1, 42) = 29.37, *p* = 0.001 Test × sex: *F*(1, 42) = 6.48, *p* = 0.001 Females > males in FT1	Sex: n.s. Treatment: n.s. Test: *F*(1, 42) = 17.12, *p* = 0.0001 FT1 > FT2	Sex: n.s. Treatment: n.s. Test: *F*(1, 42) = 55.78, *p* = 0.001 Test × treatment: *F*(2, 42) = 4.94, *p* = 0.01 OIL > BCP5, BCP10 in FT2

**Results are given per phase. See also Figure 3 for more details. n.s., not significant.**

### Step 3b: Sex Differences in the Percentage of Changes in Pain Responses Between FT1 and FT2, Repeated

**FIGURE 4:**
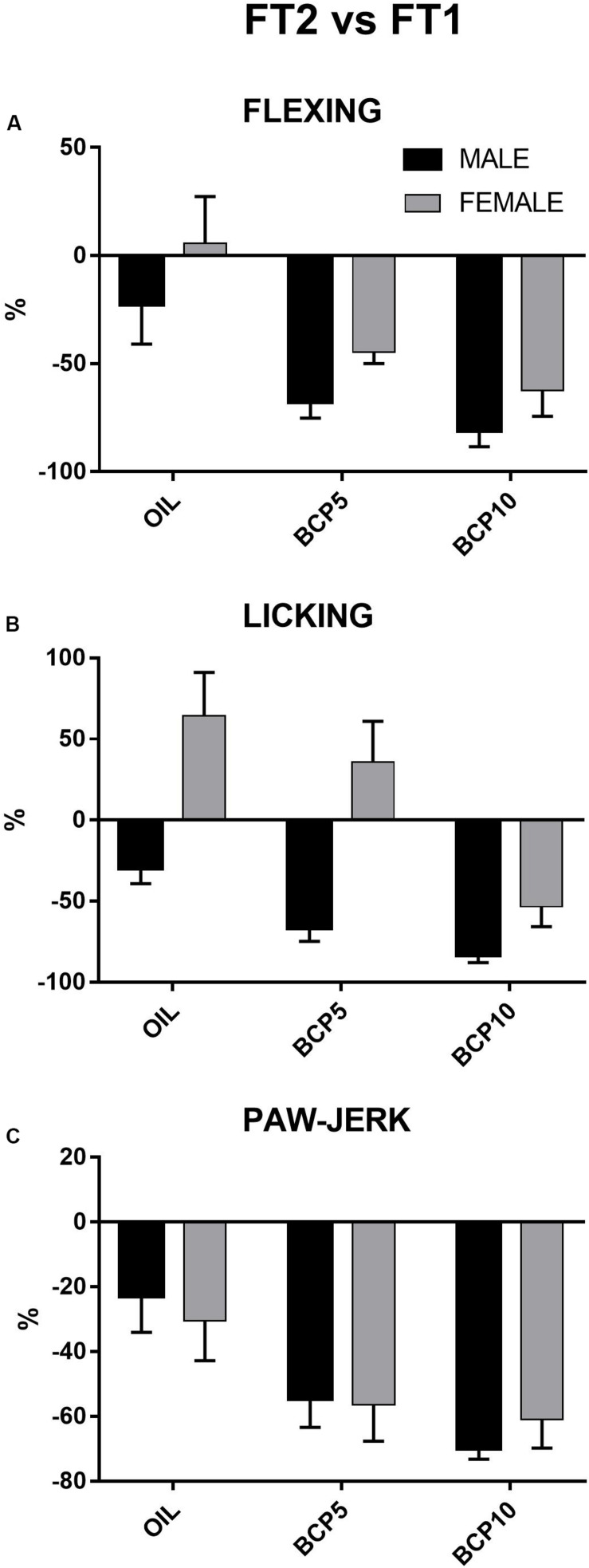
Percentages of variations of the three formalin-induced responses – flexing duration **(A)**, licking duration **(B)**, and paw jerk frequency **(C)** – between the two formalin tests (FT2 vs. FT1) in males and females: All pain responses were significantly decreased by β-caryophyllene 5 mg/kg (BCP5) and BCP 10 mg/kg (BCP10) treatments (*p* < 0.002 and *p* < 0.001 for flexing, *p* < 0.04 and *p* < 0.0001 for licking, and *p* < 0.006 and *p* < 0.001 for paw jerk). Values are the mean ± SEM.

To better represent the changes occurring in the different groups from the first to the second test, the percentages of changes of the three pain responses (flexing, licking, and paw jerk) were calculated by comparing the values of FT2 of each animal with the corresponding FT1 values. They were then subjected to two-way ANOVA with the factors treatment (three levels: OIL, BCP5, and BCP10) and sex (two levels: males, females). Independent of sex, flexing, licking, and paw jerk showed greater percentages of changes (decrease) in the BCP-treated groups than in OIL [treatment: *F*(2, 41) = 10.0, *p* = 0.002; *F*(2, 40) = 11.6, *p* = 0.0001; *F*(2, 41) = 7.9, *p* < 0.001, respectively], indicating a specific effect of treatment in these groups. Moreover, licking revealed a significant effect of the factor sex [*F*(1, 40) = 26.9, *p* = 0.001] due to females being different from males independent of groups (details are reported in Figure 4).

### Histological Examination

The absence of a significant effect of BCP treatment on the digestive system was confirmed by the lack of changes observed in the stomach and small intestine samples.

**FIGURE 5:**
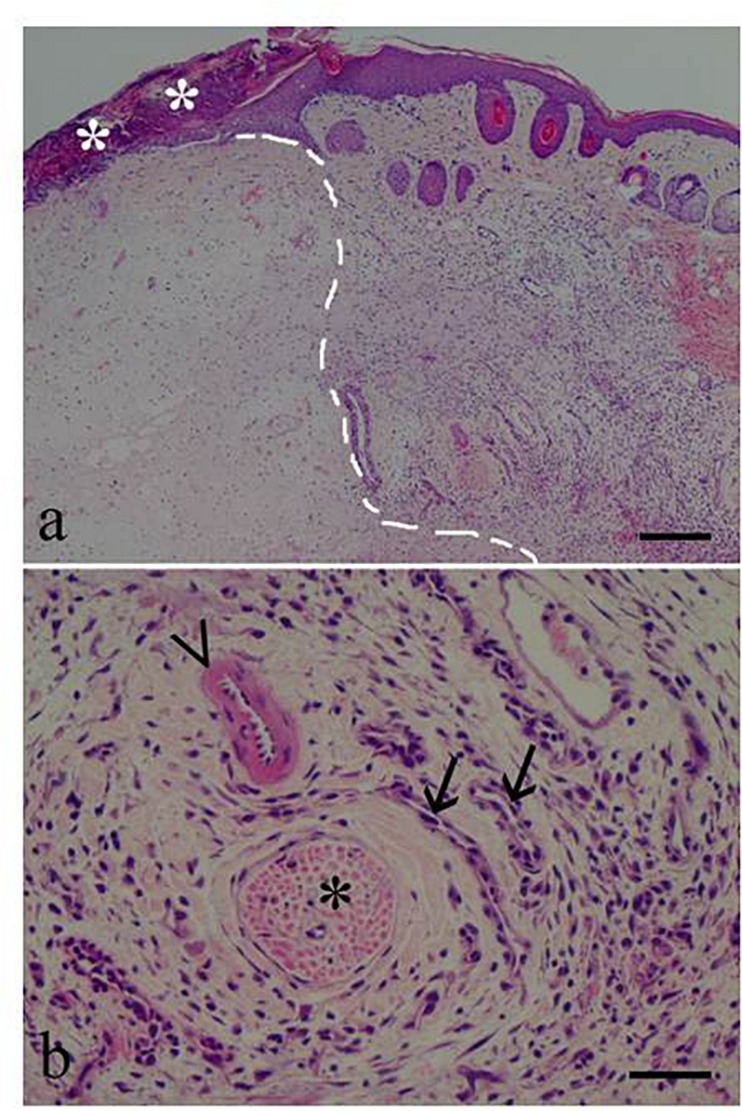
Skin photomicrographs from a male given β-caryophyllene 5 mg/kg (BCP5). **(a)** Low magnification (×40) showing the formalin injection site (*left of the dotted line*) and the inflammatory response area. *Asterisks* indicate a crust. **(b)** High magnification (×200) showing a nerve (*asterisk*), an arteriole (*arrowhead*), and two venules (*arrows*). The surrounding mononuclear inflammatory response was graded, as marked. *Scale bars*, 200 μm in **(a)** and 50 μm in **(b)**.

Skin lesions at the formalin injection site were graded as mild (low degree of edema and lymphoplasmacellular infiltrates) in BCP10 females, moderate (increased edema severity and mixed infiltrates in the majority of cases, albeit with lymphoplasmacellular infiltrates in some cases) in the OIL males and females and in BCP5 females, and marked (high degree of edema and always a mixed population of infiltrates) in the males treated with BCP5 and BCP10 (Figure 5). The skin lesions were never graded as severe.

## Discussion

The main results of the present experiment are the sex difference in the long-term effect of a nociceptive stimulation, with females showing higher/longer-lasting reactivity to repetitions of the stimulus and the strong analgesic effect of the BCP treatment in both sexes, albeit greater in males.

To mimic an animal model of recurrent pain, i.e., a common form of chronic pain during which acute short- or long-lasting episodes affect the patient for hours or days after which the symptoms disappear until the next painful events, we carried out two formalin tests separated by a 1-week period. Formalin injection causes tissue damage and inflammation involving the release of mediators from the damaged cells into the periphery. Damaged tissue will also recruit immune cells, which release cytokines and growth factors. These mediators can act directly on nociceptors to induce pain or stimulate the release of additional inflammatory agents, leading to peripheral sensitization and hyperalgesia (see Thompson et al., 1995; Kidd and Urban, 2001). Sensitization can occur in the periphery as well as in the CNS. In the CNS, astrocytes are highly involved in pain chronicization (i.e., in neuropathic pain), and they represent the majority of glial cells. In the spinal cord, astrocytes have been observed in models of neuropathic pain (Liu et al., 2000), and inhibition of such astrocyte proliferation reduced neuropathic pain (Tsuda et al., 2011). A strong sex difference was observed at this level: Sorge and Totsch (2017) showed that male mice utilize the microglia in the spinal cord to mediate pain, whereas females preferentially use T cells in a similar manner.

In the present experiment, the animals injected with 2.5% formalin in the first formalin test and not specifically treated with anti-inflammatory agents during the subsequent week (only with the vehicle, olive oil) exhibited sex differences in the three formalin-induced responses during the second test (carried out with 1% formalin). In males, the levels remained similar in FT2 with respect to FT1, with a small decrease in flexing and paw jerk and no change in licking, while in females only paw jerk decreased, flexing did not change, and licking increased significantly. As females produce a higher pro-inflammatory immune response to tissue damage than do males, it is possible that females simply developed more inflammation, directly resulting in more pain with the second injection.

Also, in the pain responses in which the levels were higher in males during FT1 (i.e., licking in the second phases), the repetition of the test induced higher levels in females, both in the group treated only with the vehicle and in the BCP-treated groups, suggesting a milder effect of BCP in females. This result can be explained by a stronger adaptive immune system in females than in males and, thus, greater immune response to injuries. Gonadal hormones appear to play an active role in these sex differences. Because of their high testosterone levels, males express the Th2 immune population in their CD4^+^ cells, whereas females express the Th1 immune population because of their lower testosterone and higher estrogen levels (Rosen et al., 2017). Circulating estrogens increase the pro-inflammatory cytokines released by mast cells, macrophages, and T cells. Since testosterone increases the macrophage production of anti-inflammatory cytokines, it is likely that males do not mount as strong an immune response to injury as do females. Thus, males could show a delay in wound healing (as in the present study) and may be less susceptible to developing a strong increase in neuronal sensitivity produced by pro-inflammatory cytokines, leading to a lower pain response than in females. This can affect not only the initiation of neuropathic pain but also its maintenance.

Here, we used a plant-derived compound to evaluate the possibility to modulate the long-term effect of a nociceptive stimulation in both male and female animals. Immediately after the first formalin test, all animals received *per os* olive oil alone or supplemented with β-caryophyllene (5 or 10 mg/kg) once a day for 1 week. β-caryophyllene is a sesquiterpene found in large amounts in the essential oils of various spice and food plants; it is also a major component (up to 35%) in the essential oil of *Cannabis sativa* (Gertsch et al., 2008). BCP has been reported to exert protective effects in experimental animal models of inflammatory pain (Gertsch et al., 2008), neurological diseases (Sharma et al., 2016), and interstitial cystitis (Berger et al., 2019).

BCP selectively binds to the CP55,940 binding site (i.e., THC binding site) in the CB2 receptor, leading to cellular activation and an anti-inflammatory effect. CB2 receptor ligands have been shown to inhibit inflammation and edema formation and thus to have an analgesic effect (Klauke et al., 2014).

In a model of neuropathic pain, the increase in spinal microglia was accompanied by an increase in CB2 receptors (Zhang et al., 2003; Romero and Orgado, 2009). Microglial activation and hyperalgesia are reduced by cannabinoid CB2 receptor agonists. This suggests that the spinal microglia could be activated and the number of CB2 receptors in the microglia increased in neuropathic pain induced by nerve injury and that the stimulation of CB2 receptors by cannabinoids inhibits the activation of the microglia. Agonists can produce analgesia *via* supraspinal, spinal, and peripheral CB receptor activation (Guindon and Hohmann, 2009).

In the present experiment, the long-lasting administration of BCP led to a strong decrease of the pain-induced responses in both sexes, with the exception of licking in females. However, it had a greater analgesic effect in males, with both BCP-treated groups showing a decrease of well over 50% with respect to FT1, even approaching 90% with the higher concentration, as shown in Figure 4.

In OIL-treated males, licking did not change significantly in the second test, in contrast to paw jerk and flexing, the most spinally mediated reflexes. However, licking was drastically decreased in males in both BCP-treated groups. In view of the literature reports on the changes in CB2 receptors in the spinal cord during long-term nociceptive stimulation and on the analgesic effects of CB2 agonists (Guindon and Beaulieu, 2009), we can state that our data support the ability of this compound to exert an analgesic effect through modulation of the cannabinoid system at both the spinal and supraspinal levels in males, whereas in females the analgesic effect appears to be limited to the spinal cord-mediated reflexes (flexing and paw jerk). Indeed, in females, the effect is not so clear. While there was a decrease in flexing and paw jerk in both treated groups, the strong increase in licking (≥50%) seen in the OIL group was not completely counteracted, although it was lower in both BCP-treated groups.

This substantial sex difference can be explained by the different circuits activated by the nociceptive and/or cannabinoid systems particularly at supraspinal levels. This is suggested by the finding in guinea pigs that cannabinoid agonists have a greater hyperphagic effect in males than in females (Miller et al., 2004; Diaz et al., 2009), indicating that cannabinoids induce greater changes in the male brain than in the female one. Nevertheless, the two cannabinoids, THC and CP55,940, were found to have twice the effect in females than in males in experimental models of phasic painful stimulation (Craft et al., 2013). Moreover, the development of tolerance to the antinociceptive and locomotor effects of THC may also be greater in females than in males (Wakley et al., 2014), and the CB1 and CB2 mRNA levels in the brain stem were also higher in female rats than in males (Xing et al., 2011). Interestingly, sex differences were found in the locomotor response to cannabinoids, with females showing less locomotor activity than do males (Craft et al., 2013).

The substantial sex differences observed in experimental animals and humans are supported by the strong responsiveness of this system to gonadal hormones, i.e., androgens and estrogens, present in both sexes, but at different levels. In adult rats, the endocannabinoid system is strongly influenced by circulating levels of estradiol (López, 2010); indeed, THC-induced antinociception is more effective in females in late proestrus–estrus (Craft and Leitl, 2008) with higher estradiol levels.

In the present experiment, all females displayed fixed estrus at the time of the second formalin test, as often occurs in a situation of chronic stress. This condition is accompanied by very low levels of estrogens, which can explain the lower BCP-induced analgesic effect in this sex. Indeed, we previously showed that while (low) physiological levels of estrogen “help” females to feel pain, higher supra-physiological levels act as an analgesic (Aloisi et al., 2010). Moreover, high estrogen levels were found to activate the endorphinergic system in humans (Smith et al., 2006) and to reduce the astrocyte numbers in the spinal cord of chronic constriction injury (CCI) mice (Vacca et al., 2016); thus, it appears that this system cannot be fully active in this condition, i.e., there is a lower analgesic effect due to the lower estrogen levels available.

## Conclusion

We have confirmed the higher reactivity of females to prolonged inflammatory stimulation, and we have demonstrated the important analgesic role of the CB2 agonist β-caryophyllene in both sexes.

## Data Availability Statement

All datasets presented in this study are included in the article/supplementary material.

## Ethics Statement

This study received the approval of the Local Ethics Committee (Aut. Min 65/2011B).

## Author Contributions

IC, PF, FP, JP, MA, VM, and AA: project and execution of the experiment. IC and JP: statistical analysis. IC, FP, and AA: writing the manuscript. All authors contributed to the article and approved the submitted version.

## Conflict of Interest

The authors declare that the research was conducted in the absence of any commercial or financial relationships that could be construed as a potential conflict of interest.
